# “Happy feet”: evaluating the benefits of a 100-day 10,000 step challenge on mental health and wellbeing

**DOI:** 10.1186/s12888-018-1609-y

**Published:** 2018-01-24

**Authors:** K. T. Hallam, S. Bilsborough, M. de Courten

**Affiliations:** 1 Centre for Chronic Disease, College of Health and Biomedicine, Victoria University, Melbourne, Australia; 2 Outlook Matters, Pty Ltd, Melbourne, Australia; 3 Stepathlon Private Limited, No. 2B 25E, Lorette Ville, Ground Floor, Village Bandra, Santacruz West, Main Avenue, Mumbai, India

**Keywords:** Depression, Stress, Anxiety, Wellbeing, Physical activity, Health promotion

## Abstract

**Background:**

An increased awareness of the health benefits of walking has emerged with the development and refinement of accelerometer equipment. Evidence is beginning to highlight the value of promoting walking, particularly focusing on the Japanese mark of obtaining 10,000 steps per day. Workplace based step challenges have become popular to engage large cohorts in increasing their daily physical activity in a sustainable and enjoyable way. Findings are now highlighting the positive health effects of these medium-term programs (typically conducted over a few months) in terms of cardiovascular health, reducing diabetes risk and improving lifestyle factors such as weight and blood pressure. As yet, research has not focused on whether similar improvements in psychological health and wellbeing are present.

**Methods:**

This study investigated the impact of a 100-day, 10,000 step program on signs of depression, anxiety and stress as well as general wellbeing using standardised psychological scales.

**Results:**

The results indicated a small but consistent effect on all of these measures of mental health over the term of the program. This effect appeared irrespective of whether a person reached the 10,000 step mark.

**Conclusions:**

These results highlight improved mental health and wellbeing in people undertaking this 100-day 10,000 step program and indicates the efficacy and potential of these programs for a modest, yet important improvement in mental health. Notably, targets reached may be less important than participation itself.

## Background

By 2030, mental illness is projected to cost the global community over US$ 6 T [1]. This places mental illnesses impacts above cardiovascular disease, chronic respiratory disease, cancer and diabetes [1]. People experiencing mental illness are also known to be at a much higher risk for morbidity and early mortality from physical health issues than the general population [2]. This risk may be further exacerbated by the medications utilised in the treatment of these illnesses [2, 3]. Associated with this high treatment cost is the emerging equity and access issue in low income countries associated with psychiatric disability or sub syndromal functional decline. In these contexts, 75%–85% of people with mental health issues are untreated [4]. Paired with a shortfall of mental health specialists in low and middle income countries [5], these data point to the importance of primary prevention and population level interventions to improve mental health and wellbeing. Physical activity (PA) has become an increasingly important focus in the prevention of non-communicable diseases, yet these strategies are often underutilised in the mental illness (and mental health) domain [6–8]. Epidemiological studies demonstrate that adequate PA is associated with fewer depressive symptoms [9], and conversely, the development of depressive symptoms many result from insufficient PA [10]. Four Cochrane Database Systematic reviews, titled “Exercise and Depression”, have been undertaken in the past 10 years. The most recent of these meta-analyses showed that in clinically depressed individuals, exercise has a moderate effect on symptoms of depression, highlighting its place as an adjunct treatment option [11].

Physical activity likely exerts its impact on mood via reducing the activity of the sympathetic nervous system and associated hypothalamic-pituitary-adrenal axis reactivity in the brain [12]. In terms of monoaminergic activity, animal models highlight the role of exercise in increasing both serotonin and noradrenalin levels in the brain in a similar fashion to antidepressant medications [13]. On a clinical level, this serotonergic activity may explain why exercise is as effective as serotonergic focused antidepressant medications for mild to moderate symptoms [14]. Evidence also indicates increased brain derived neurotrophic factor (BDNF) levels associated with single sessions of exercise that improve further with regular exercise. This increased BDNF level is associated with both cognitive and mood improvements in the brain and behaviour [15]. Smits and colleagues (2008) have demonstrated that with as little as six, 20 min sessions of treadmill exercise (at 70% maximum health rate) over two weeks has a positive impact on mood factors such as anxiety [16]. It is clear that promoting PA for both clinical and sub-clinical mood disturbance is a valuable goal.

Stress is a precipitating factor in susceptibility and exacerbation of mental health issues. Stress is more commonly experienced than depression and anxiety and is associated both with poorer mental health and wellbeing, an important linkage as wellbeing itself is associated with better physical and psychological health over the lifespan [17]. Notably, stress is very responsive to PA [18] and regularly engaging in PA has been recommended by many government and health authorities for improving mental and physical health and wellbeing [19, 20].

The World Health Organisation [21] has identified a valuable role for workplace based interventions in improving the health of the whole workforce. Pedometer based programs can significantly improve physical activity and physical health including reducing sitting time, reducing weight, improving blood lipid levels and reduced diabetes risk indicators [22–24]. Glance and colleagues [24] have also indicated an improvement in wellbeing, implying an impact on stress levels in participants. Within step challenge programs, the 10,000 step goal roughly equate to 8 km of walking per day (around 1.5 to 2 h of walking). This level of activity well exceeds the WHO Global Strategy on Physical Health, Diet and Activity recommendations of 150 min of moderate physical activity per week [25]. Despite clear physical improvements associated with 10,000 step programs, to date no research has investigated possible important impacts of these workplace programs or the 10,000 daily step benchmark on the psychological factors of wellbeing, stress, anxiety and depression. Whether 10,000 steps are sufficient to elicit changes in these (perhaps more complex) psychological domains remains unknown at this time [26].

The aim of this investigation was to assess the impact of a 100-day workplace based 10,000 step challenge on mental health and wellbeing in an international workplace community sample. Based on evidence indicating positive physical health effects from 10,000 step programs four hypotheses were developed. Firstly, it was hypothesised that participants involved in a 10,000 step program for 100 days would see benefits in their levels of depression, anxiety and stress and consequent improvements in wellbeing scores. Further, it was hypothesised that increasing amounts of exercise in these programs would lead to improved outcomes on these. Thirdly it was hypothesised that those who on average completed more than 10,000 steps per day throughout (the recommended level for these programs) would perform better in terms of mental health and wellbeing than those who did not reach this threshold. Finally, it was hypothesised that when controlling for demographic factors of age and gender, this improved outcome in people who engaged in more stepping exercise would be maintained.

## Methods

### Participants

Of the 2379 respondents who consented to participate in this research, 1963 participants made up the final sample size. This number accounted for 416 who either did not complete 70% of the 100-day challenge or those who did not complete the demographic or mental health survey.

### Materials

Stepathlon is a company headquartered in Mumbai, India running globally organized team-based walking programs at participating workplaces. Stepathlon has also offices in Australia where the workplace health and wellbeing policies are developed. Hence, the study sample are predominantly from these two countries.

Through the Stepathlon corporate challenge (http://www.stepathlon.com), routine data are obtained relating to age, gender, location and daily step counts as recorded by participants on a password protected website based on readings from pedometer (clip-on) or other fitness monitors (e.g. wrist accelerometer). On completion of the 100-day step challenge in 2015 and 2016, digital assets management staff of Stepathlon extracted de-identified data from their server and provided this de-identified database to Victoria University as per research agreement.

Symptoms of depression, anxiety and stress were assessed using the short form of the Depression, Anxiety Stress Scales (DASS [27]). The short form DASS is a 21-item, self-report, symptom-oriented measure consisting of three sub scales: Depression, Anxiety and Stress. Participants rated their experience of each indicator across a four-point frequency (from 0 [did not apply to me at all] to 3 [applies very much to me or most of the time]). The DASS has strong construct validity [28], adequate discriminant and convergent validity [27] and internal consistency (Cronbach’s α of 0.96, 0.89, 0.93 for the depression, anxiety and stress subscales respectively [29]). It has strong factor structure in both non-clinical [27] and clinical samples [29].

The Warwick-Edinburgh Mental Wellbeing Scale (WEMWBS) is a 14-item measure rated on a 1–5 scale (from ‘none of the time’ to ‘all of the time’). The scale focuses on positive elements of wellbeing and is positively worded throughout. The scale provides a one factor model for measuring wellbeing [30, 31]. The WEMWBS shows strong criterion validity with the WHO Wellbeing Index-5, Positive and Negative Affect Schedule and is negatively correlated with the General Health Questionnaire-12 measure of mental ill health [30]. Finally, internal consistency (Cronbach’s α = 0.91) and one-week test-retest reliability (0.83) of the scale were strong [30].

### Procedure

Participants were recruited from the established international Stepathlon program. This workplace activity challenge was promoted at participating sites via posters, e-flyers, and through engagement of senior management through visits to sites by health & wellbeing managers.

Participants of this workplace activity challenge were provided with a participant information and consent forms inviting them to this research charting the impacts of the 10,000 step challenge on their mental health and wellbeing. The project was approved by the Victoria University Human Research Ethics Committee (HRE15–168). All participants completed the standard demographics questionnaire and logged their daily step count for as many of 100 days during the challenge. Participants could use their own pedometer, or activity monitoring device throughout the challenge and recorded their daily totals via the Stepathlon user portal. Through this portal they received regular newsletters, updates and motivational articles for the duration of the challenge. In addition to the standard step challenge package, consenting participants completed a pre-and post 100-day challenge questionnaire regarding their mental health using the DASS-21 and WEMWBS questionnaires in English.

### Design

The results of the study were analysed using a number of repeated measures ANOVAs. Firstly, the data were assessed to see if there were overall changes in mood and wellbeing from pre to post testing over the activity challenge. Following this, a repeated measures ANOVA focusing on the 10,000 step threshold (between subjects factor) and change over time (within subjects factor) was used to evaluate the 10,000 step criterion. Further, a series of Pearson product moment correlation coefficients were utilised to assess overall relationships between number of steps completed on average by participants and changes in mental health and wellbeing scores. Finally, a repeated measures ANCOVA was utilised to assess the factors in the 10,000 steps over time but covarying for age and gender. All analysis was conducted in SPSS version 21.

## Results

The final sample of 1963 participants was screened for univariate outliers via box plot analysis on the outcome scales. Data inspection indicated no outliers (+ 2 SD) in the sample, presumably due to the large sample size. Data were assessed for the assumptions of normality and skewness. The data did not violate the assumptions of asymmetry and kurtosis of ±2 using threshold defined by Field [32]. Tests of within subject violations of the assumption of sphericity indicated no violations of this measure, hence parametric analysis was appropriate for the within subjects analysis. Finally, violation of the assumption of homogeneity was assessed using Levene’s equality of error variance estimates. Due to the large sample size in the study, the Levene’s significant level was adjusted to 0.001 as per the recommendations of Warner [33]. The results after correction showed no violations of homogeneity of variance, hence parametric analysis was used for all statistical analysis.

### Demographics

The final sample represented 1458 males and 505 females who ranged in age from 16 to 74 years (M = 36.6, SD = 8.9). Participants represented numerous countries of origin with the majority being from India (*n* = 1610) and Australia (*n* = 227) along with 21 other countries.

### Change in mental health and wellbeing over 100 day challenge

A repeated measures ANOVA demonstrated improvements on all measures of the DASS over the 100-day challenge period. This included a significant 7.6% improvement in levels of depressive symptoms based on a reduction from an average of 4.59 (S.D. = 3.52) to 4.24 (S.D. = 3.44), *F*(1, 1962) = 67.385, *p* < .001, *η*^*2*^ = .033, *power* = 1.0. In relation to anxious symptoms, the data showed a 5.04% improvement after the 100 day challenge, reducing from an average of 4.56 (S.D. = 3.13) to 4.33 (S.D. = 3.08), *F*(1, 1962) = 34.416, *p* < .001, *η*^*2*^ = .0017, *power* = 1.0. Stress symptoms were the most responsive to the 100-day challenge with the data showing an 8.96% improvement in stress levels on the DSS following the program. This equated to a reduction from an average of 5.47 (S.D. = 3.19) to 4.98 (S.D. = 3.05), *F*(1, 1962) = 117.593, *p* < .001, *η*^*2*^ = .057, *power* = 1.0. Finally, there was a 2.13% improvement in overall wellbeing on the WEMWBS, associated with an increase in average wellbeing scores from 50.01 (S.D. = 10.29) to 51.08 (S.D. = 10.45), *F*(1, 1962) = 103.555, *p* < .001, *η*^*2*^ = .050, *power* = 1.0.

### Relationship between mental health and wellbeing score changes and average amount of walking

To investigate if these clear mental health improvements over the challenge were associated with increasing levels of walking exercise a series of correlation analyses were conducted contrasting change scores on each of these measures from pre to post testing versus the average number of steps taken per day. The results indicated no significant overall correlations between average steps and depression (*r*^*2*^ = − 0.026, *p* = .254), anxiety (*r*^*2*^ = −.029, *p* = .205), stress (*r*^*2*^ = −.026, *p* = .247) and WEMWBS (*r*^*2*^ = .026, *p* = .256). While all measures trend in the direction of improvement with increasing steps, it is notable that the range (2775 average steps to 112,831 average steps) of steps taken on average varied markedly, impacting clear correlations.

### Do people who walk over 10,000 steps on average have greater improvements in mental health and wellbeing?

It is contended in 10,000 step programs that reaching 10,000 steps has a certain significance for participants. This “stepping goal” was assessed to elucidate any meaningful differences between those who reached less than or greater than this goal on average across the program. This research question was assessed with a repeated measures ANOVA that compared the between subject factor of number of steps (less than 10,000 versus equal to or more than 10,000 steps). These results are depicted in Table 1.

**Table 1:** Change in mental state and wellbeing scores across 100-day challenge based on participant’s average 10,000 step goal

	Depression		Anxiety		Stress		WEMWBS	
	Pre	Post	Pre	Post	Pre	Post	Pre	Post
< 10,000 steps (*n* = 435)	4.72(0.17)	4.49(0.16)	4.47(0.15)	4.41(0.15)	5.97(0.16)	5.65(0.15)	49.30(0.47)	50.08(0.47)
≥ 10,000 steps (*n* = 1528)	4.55(0.10)	4.17(0.09)	4.58(0.08)	4.31(0.08)	5.33(0.08)	4.79(0.08)	50.22(0.27)	51.37(0.27)
Change over time	*F*(1, 1961) = 62.462, *p* < .001, *η*^*2*^ = 0.031		*F*(1, 1961) = 12.831, *p* < .001, *η*^2^ = 0.007		*F*(1, 1961) = 62.462, *p* < .001, *η*^2^ = 0.031		*F*(1, 1961) = 58.47, *p* < .001, *η*^2^ = 0.029	

The results highlighted an interaction effect between group (less than 10,000 steps versus 10,000 or more steps) and time (pre and post testing) for DASS stress (*F* [1, 1961] = 3.982, *p* = .046, *η2* = .002, *power* = .514) with the participants who completed equal to or more than 10,000 steps showing a 10.13% improvement in stress levels whilst the individuals who completed less than 10,000 steps daily only achieved a 5.36% improvement.

Likewise, the interaction effect around anxiety showed that those who completed equal to or more than 10,000 steps daily had a significantly better improvement in anxiety over the challenge, improving their anxiety levels by 5.89% versus only 1.34% for those who completed less than 10,000 steps. (*F* [1, 1961] = 5.421, *p* < .001, *η2* = .003, *power* = .643). Interaction effects for depression and overall wellbeing scores were not evident in the analysis. Finally, between subjects differences profiling overall levels of psychological distress and wellbeing in those who did less than 10,000 steps on average versus those who did more showed that the participants in the greater than 10,000 steps group had better scores on wellbeing, *F*(1, 1961) = 4.017, *p* = .045, *η2* = .002, *power* = .517, and stress levels, *F*(1, 1961) = 21.795, *p* < .001, *η2* = .011, *power* = .997. There were no overall differences in anxiety, *F*(1, 1961) = .001, *p* = .975, *η2* = .000, *power* = .050 and depression scores, *F* (1, 1961) = 1.876, *p* = .171, η2 = .001, *power* = .278.

### Do 10,000 step differences remain when age and gender are accounted for?

A final analysis was conducted to see if adding demographic factors other than the 10,000 steps model impacted the importance of meeting this threshold in relation to mental health and wellbeing. Gender was divided into male and female participants whilst age was categorised as 18–30 years (*n* = 519), 31–45 years (1096), 46–60 years (*n* = 292) and 60 plus (*n* = 24 years). This analysis continued to use pre and post-depression/anxiety/stress and WEMWBS levels as the within subjects factors and now included threshold (i.e. more or less than 10,000 steps achieved on average) as the between subjects factor as above. Gender and age were covariates in the model.

The ANCOVA analysis of depression continued to reflect no significant change in depression levels over time (within subjects factor) whilst gender and age category were both significant between subjects predictors of depression scores overall, *F* (1, 1927) =13.402, *p* < .001, *eta*^*2*^ = .007, *power* .955 and *F* (1, 1927) =22.725, *p* < .001, *eta*^*2*^ = .012, *power* = .997 respectively. Whether people had reached 10,000 steps daily on average had no added significant effects. Finally, there were no interaction effects with the between subjects covariates and the 10,000 step threshold factor and change in depression levels over time.

The ANCOVA analysis of anxiety again revealed the overall decrease in anxiety over the challenge but no significant interactions between age, gender or the 10,000 step threshold in the changes observed in the within subjects analysis. In terms of between subjects effects of the threshold measure and the covariates of age and gender, the results highlight an overall impact of the age covariate, *F*(1,1927) = 52.363, *p* < .001, *eta*^*2*^ = .026, *power* = 1 but no significant impacts from whether people achieve the 10,000 step threshold or their gender.

The ANCOVA related to stress highlighted that with the fuller model, the former significant change over time was no longer observable. Further, there were no significant interaction effects in relation to stress changes for whether people reached the 10,000 step threshold or related to the covariates age and gender. The between subjects analysis highlighted that age and whether people walked over 10,000 had significant impacts on stress levels. in terms of overall stress levels, both age and gender impacted, *F*(1,1927) = 13.707, *p* < .001, *eta*^*2*^.007, *power* = .959 and *F*(1,1927) = 23.616, *p* < .001, *eta*^*2*^ = .012, *power* = .998. Notably, the participants who were in the group that had averaged over 10,000 steps were those with lower stress levels overall.

Finally, wellbeing scores were assessed. The data from the WEMWBS indicated that the wellbeing scores were unchanged by participation in the program overall and there were no significant interactions between other factors and the results on this scale. Both covariates showed impacts on the between subjects results of the WEMWBS including those for gender, *F*(1,1927) = 56.608), *p* < .001, *eta*^*2*^ = .004, *power* = 1.0 and age *F*(1,1927)7.315, *p* = .007, *eta*^*2*^ = .004, *power* = .771. Notably, being in the above the 10,000 step threshold group did not infer any differences.

## Discussion

The results of this study highlight some psychological and wellbeing benefits of being engaged in work based 10,000 step programs. This effect appeared irrespective of the average number of steps achieved by the person over the program period. Mental health is related to a range of biopsychosocial factors and as a result a range of interventions and approaches may make up sustaining good mental health. This study demonstrated that engaging in a workplace based step program improved stress levels by 8.9%, signs of depression by 7.6%, anxiety by 5.0% and wellbeing by 2.1% from baseline. This reinforces the benefits of this type of exercise regimen as playing a small yet significant role in improving mental as well as physical health. It is notable that when considering the role of the 10,000 step threshold as a significant number to improve people on these psychological factors, there is little evidence from this study that this number holds any significant value. Further, evidence indicates that even the most basic demographic variables such as gender and age can impact the outcomes of this number of steps, again reinforcing the complex biopsychosocial milieu that makes up mental health and wellbeing.

The lack of a dose response highlights there may be multiple factors that lead to improvements associated with these programs outside activity level per se. This may include the enjoyment and connection obtained from meaningful group participation, teamwork, improved friendship, interpersonal support and networking that has been shown to significantly improve mental health and wellbeing [34]. Further, health promoting activities often lead to other behavioural change (e.g. improved diet) to potentially further improve outcomes. The goal of these public health campaign approaches is exactly this, the creation of a gestalt (i.e. the end result being more than just the parts, e.g. doing 10,000 steps specifically) of external and internal change to improve both individual and collective group health. Further research into this cohort needs to explore which combination of internal and external factors most predict successful behavioural change in work based programs. A limitation of this study is the lack of follow-up after the program has ceased to see if mental health gains and behavioural change have been maintained or if they only exist within the workplace challenge. Put plainly, 10,000 steps is not a magic number for the gains in mental health and wellbeing we observed but being part of a health program is clearly positive for mental health and wellbeing by the end of a program.

This workplace health challenge highlights the mental health benefits in a similar way to physical health benefits of these programs. This shows a greater impact than previously demonstrated. Mental health and wellbeing issues play a significant part in absenteeism, reduced productivity and overall decreased quality of life for many individuals. Cost savings for workplaces in terms of mental health are evident with these programs. In combination with other workplace mental health initiatives, these may contribute to the suite of different resources and approaches that improve mental health for those with sub threshold concerns and potentially some benefit for those with more serious mental health issues. This spectrum also requires further investigation.

Evidence indicates that workplace based health promotion programs reduce absenteeism and organisational health care expenditure as described in a systematic review of interventions in large US based firms with an average program duration of 3 years: there the average employee health care costs fell by US$ 3.3 for every 1$ spent on employee wellness programs [35]. However, the real value of workplace wellness programs should include outcomes beyond financial or economic factors, such as physical and mental health, quality of life and other, non-health related factors that improve employee wellbeing, satisfaction and reduce turnover [36].

Our research benefited from a large sample size of participants across a number of countries to fully assess the hypotheses. In terms of limitations, this study did not record diagnoses of mental health issues (including depression and anxiety) which would further elucidate the impact of the program on clinical levels of distress. Secondly, the research did not report on the individual’s overall level of activity. This may have been an important consideration as there are clearly individuals who were more active before commencing the program. Ideally this would be co-varied in future studies. Further, all activity levels are self-report and, as participants were involved in the challenge as teams, the data may reflect social desirability biases. Finally, longitudinal follow up of whether people continue to exercise and experience mental health benefits after participating in a workplace based activity challenge needs to be explored to ascertain the sustainability and return of investments of such workplace wellbeing programs. An eight-months follow-up evaluation after a comparable 4-months pedometer-based workplace health program has demonstrated sustained improvement in chronic disease risk factors indicating that such programs can have a long-term benefit [37].

## Conclusions

The increasing prevalence of non-communicable diseases such as mental ill health has heightened the need to change lifestyle behaviours and increase physical activity. With most adults spending at least half of their life working, the workplace is an important setting for promoting mental health and wellbeing change [38]. The results of this research indicate that 10,000 step challenges may significantly and meaningfully improve mental health and wellbeing through simple and inexpensive workplace based interventions.

## Abbreviations

ANCOVA: Analysis of covariance
BDNF: Brain derived neurotrophic factor
DASS: Depression, Anxiety and Stress Scales
PA: Physical Activity
WEMWBS: Warwick-Edinburgh Mental Wellbeing Scale
WHO: World Health Organization

## References

[1] Bloom D, Cafiero E, Jané-Llopis E, Abrahams-Gessel S, Bloom L, Fathima S, et al. The global economic burden of noncommunicable diseases. Geneva: World Economic Forum; 2011.

[2] De Hert M, Correll CU, Bobes J, Cetkovich-Bakmas M, Cohen D, Asai I (2011). Physical illness in patients with severe mental disorders. I. Prevalence, impact of medications and disparities in health care. World Psychiatry.

[3] Correll CU, Detraux J, De Lepeleire J, De Hert M (2015). Effects of antipsychotics, antidepressants and mood stabilizers on risk for physical diseases in people with schizophrenia, depression and bipolar disorder. World Psychiatry.

[4] Funk M, Drew N, Knapp M (2012). Mental health, poverty and development. J Public Mental Health.

[5] Kakuma R, Minas H, van Ginneken N, Dal Poz MR, Desiraju K, Morris JE (2011). Human resources for mental health care: current situation and strategies for action. Lancet.

[6] Booth FW, Chakravarthy MV, Gordon SE, Spangenburg EE (2002). Waging war on physical inactivity: using modern molecular ammunition against an ancient enemy. J Appl Physiol.

[7] Callaghan P (2004). Exercise: a neglected intervention in mental health care?. J Psychiatr Ment Health Nurs.

[8] Stanton S, Rosenbaum S, Kalucy M, Reaburn P, Happell B (2015). A call to action: exercise as treatment for patients with mental illness. Aust J Prim Health.

[9] Lucas M, Mekary R, Pan A, Mirzaei F, O'Reilly EJ, Willett WC (2011). Relation between clinical depression risk and physical activity and time spent watching television in older women: a 10-year prospective follow-up study. Am J Epidemiol.

[10] Hiles SA, Lamers F, Milaneschi Y, Penninx BWJH (2017). Sit, step, sweat: longitudinal associations between physical activity patterns, anxiety and depression. Psychol Med.

[11] Cooney GM, Dwan K, Greig CA, Lawlor DA, Rimer J, Waugh FR, Mead GE (2013). Exercise for depression. Cochrane database Syst rev.

[12] Rimmele U, Zellweger BC, Marti B, Seiler R, Mohiyeddini C, Ehlert U (2007). Trained men show lower cortisol, heart rate and psychological responses to psychosocial stress compared with untrained men. Psychoneuroendocrinology.

[13] Meeusen R, De Meirleir K (1995). Exercise and brain neurotransmission. Sports Med.

[14] Kvam S, Kleppe CL, Nordhus IH, Hovland A (2016). Exercise as a treatment for depression_ a meta-analysis. J Affect Disord.

[15] Szuhany KL, Bugatti M, Otto MW (2015). A meta-analytic review of the effects of exercise on brain-derived neurotrophic factor. J Psychiatr Res.

[16] Smits JAJ, Berry AC, Rosenfield D, Powers MB, Behar E, Otto MW (2008). Reducing anxiety sensitivity with exercise. Depress Anxiety.

[17] Steptoe A, Deaton A, Stone AA (2015). Subjective wellbeing, health, and ageing. Lancet.

[18] Salmon P (2001). Effects of physical exercise on anxiety, depression, and sensitivity to stress: a unifying theory. Clin Psychol Rev.

[19] Brown WJ, Bauman AE, Bull FC, Burton NW. Development of evidence-based physical activity recommendations for adults (18–64 years): report prepared for the Australian Government Department of Health, august 2012. Canberra: Commonwealth of Australia; 2013.

[20] Fletcher GF, Balady G, Blair SN, Blumenthal J, Caspersen C, Chaitman B, et al. Statement on exercise: benefits and recommendations for physical activity programs for all Americans. A statement for health professionals by the committee on exercise and cardiac rehabilitation of the council on clinical cardiology, American Heart Association. Circulation. 1996;94:857–62. 10.1161/01.cir.94.4.857 8772712

[21] Proper K, van Mechelen W (2008). Effectiveness and economic impact of worksite interventions to promote physical activity and healthy diet.

[22] Faghri PD, Omokaro C, Parker C, Nichols E, Gustavesen S, Blozie E (2008). E-technology and pedometer walking program to increase physical activity at work. J Prim Prev.

[23] Ganesan AN, Louise J, Horsfall M, Bilsborough SA, Hendriks J, McGavigan AD (2016). International mobile-health intervention on physical activity, sitting, and weight. J Am Coll Cardiol.

[24] Glance DG, Ooi E, Berman Y, Glance CF, Barrett HR (2016). Impact of a digital activity tracker-based workplace activity program on health and wellbeing. DH ‘16.

[25] Alwan A. Global status report on noncommunicable diseases 2010. Geneva: World Health Organization; 2011.

[26] Otto MW, Church TS, Craft LL, Greer TL, Smits JAJ, Trivedi MH (2007). Exercise for mood and anxiety disorders. Prim Care Companion J Clin Psychiatry.

[27] Lovibond SH, Lovibond PF (1995). Manual for the depression anxiety stress scales.

[28] Crawford JR, Henry JD (2003). The depression anxiety stress scales (DASS): normative data and latent structure in a large non-clinical sample. Br J Clin Psychol.

[29] Brown TA, Chorpita BF, Korotitsch W, Barlow DH (1997). Psychometric properties of the depression anxiety stress scales (DASS) in clinical samples. Behav Res Ther.

[30] Tennant R, Hiller L, Fishwick R, Platt S, Joseph S, Weich S (2007). The Warwick-Edinburgh mental well-being scale (WEMWBS): development and UK validation. Health Qual Life Outcomes.

[31] Bass M, Dawkin M, Muncer S, Vigurs S, Bostock J (2016). Validation of Warwick-Edinburgh mental well-being scale (WEMWBS) in a population of people using secondary care mental health services. J Ment Health.

[32] Field A, Carmichael M (2013). Discovering statistics using IBM SPSS statistics.

[33] Warner RM. Applied statistics: from Bivariate through multivariate techniques. Thousand Oaks: SAGE; 2012.

[34] LaMontagne AD, Martin A, Page KM, Reavley NJ, Noblet AJ, Milner AJ (2014). Workplace mental health: developing an integrated intervention approach. BMC Psychiatry.

[35] Baicker K, Cutler D, Song Z (2010). Workplace wellness programs can generate savings. Health Aff.

[36] Pronk NP (2014). Placing workplace wellness in proper context: value beyond money. Prev Chronic Dis.

[37] Freak-Poli R, Wolfe R, Brand M, de Courten M, Peeters A (2013). Eight-month postprogram completion: change in risk factors for chronic disease amongst participants in a 4-month pedometer-based workplace health program. Obesity (Silver Spring).

[38] Conn VS, Hafdahl AR, Cooper PS, Brown LM, Lusk SL (2009). Meta-analysis of workplace physical activity interventions. Am J Prev Med.

